# Secretory Vesicle and Glucoamylase Distribution in *Aspergillus niger* and Macromorphology in Regions of Varying Shear Stress

**DOI:** 10.3389/fmicb.2022.842249

**Published:** 2022-05-20

**Authors:** Philipp Kunz, Rudibert King

**Affiliations:** Chair of Measurement and Control, Technische Universität Berlin, Berlin, Germany

**Keywords:** *A. niger*, flow chamber, backward-facing step, shear stress, morphology, secretory vesicles, glucoamylase

## Abstract

In technical fermentations, filamentous microorganisms are exposed to different forms of mechanical stress, among which shear stress is prevalent in turbulent broths. Whereas small-scale bioreactors allow for realistic turbulent flow field conditions, they are not well-suited to investigate the fungal response to shear stress in more detail, as they only reveal the integral effect of a highly dynamic stress stimulus. Therefore, the widely used model system for producing constant, but rather low shear forces, the parallel plate flow chamber, is extended in this work by adding a backward-facing step (BFS). The BFS induces vortex shedding in the wake of the step and brings out distinct areas of different shear stress levels at the bottom of the chamber where mycelia grow. This allows for a stress-dependent analysis of growing cells using a confocal laser-scanning microscope. As the real stress cannot be measured in the experiment, the wall shear stress is estimated numerically using computational fluid dynamics (CFD). As a first application of the experimental setup, the relative biomass concentration, the relative amount of secretory vesicles and the relative amount of the chosen product glucoamylase produced by the filamentous fungus *Aspergillus niger* were measured. The obtained area scans show homogeneous mycelia growth in areas of low stress and cloud-like patterns downstream of the predicted flow reattachment length where high shear stress dominates. Quantitative analysis of the time course suggests that the amount of available secretory vesicles inside of *A. niger* decreases when the shear stress is increased, despite that no significant differences in biomass production could be found. In contrast, the highest level of glucoamylase was reached for intermediate volumetric flow rates, i.e., levels of shear stress.

## 1. Introduction

Hydromechanical stress is considered a decisive influencing variable for multiphase processes in the field of bioprocess engineering. In production-scale fermentations, stirring or mixing processes are often necessary to ensure adequate oxygen supply to and CO_2_ romoval from the growing organisms, to support heat transfer, and to homogenize the broth. The hydrodynamic conditions created during cell culture in bioreactors can critically affect morphology, cell metabolism, and production capacities (Kelly et al., 2004; Serrano-Carreón et al., 2015). More specifically, as one of many open questions formulated in the challenging primer paper by Meyer et al. (2021), it was asked whether enhanced shear stress changes the hyphal distribution of intracellular vesicles, which are needed both for length growth and product secretion, and how such a modified shear stress can be produced and studied on a microscopic scale. This contribution will present first results in that direction from a more macroscopic point of view, i.e., by low microscopic magnification.

During liquid culture fermentation, fungal components experience various types of mechanical stress, including impact and shear forces. The former results from density differences between the liquid and the particles or contact stress occurring between individual particles, particles and the stirrer, or between particles and the reactor wall (Henzler, 2000). Particles can be individual biological entities of varying sizes, for example fungal spores or pellets. If the density difference between those and the liquid is negligible, the shear forces dominate. This means that the stress these particles experience is mainly the result of the relative velocity between the particles and the surrounding fluid. High shear forces can lead to morphological changes such as rupture or lysis, especially for shear-sensitive cells. Since the density of fungal mycelia differs only very slightly from the density of the surrounding medium, fungal components in agitated media mainly follow the streamlines of the liquid. Hence, the interaction of *A. niger* pellets with fluid vortices is the main cause of fragmentation in stirred processes (Kelly et al., 2004). Local shear gradients are associated with increased fracture probability of particles in stirred reactors (Maaß et al., 2009).

Quantifying the shear forces acting on individual hyphae during real reactor cultivation is challenging for several reasons. Bioreactors are complexly shaped vessels characterized with or without moving components (e.g., stirrers), flow breakers, and dead zones, i.e., highly dynamic velocity fields must be expected. Fermentation broths of filamentous organisms are heterogeneous multi-phase mixtures that can exhibit local accumulations of biomass, high viscosity, and non-Newtonian flow behavior. Inherent to the fermentation in reactors, the individual sources of stress are difficult to quantify and isolate during reactor operation. According to Henzler (2000), stress on particles becomes evident only as an integral result of a long-term process, i.e., the fermentation. Classically integral quantities such as the global shear rate, stirrer speed, power input, rate of energy dissipation, or other surrogates are used to quantify mixing and energy input (Kelly et al., 2004; Krull et al., 2010). Average shear rates experienced in lab-scale fermenters in literature vary between $\dot{\gamma }=1-1,000$ 1/s for stirrer rotational speeds of 200–600 1/min (Bliatsiou et al., 2020; calculated by Metzner-Otto approach, therefore assuming stirred tank under laminar flow). However, the corresponding estimates should be treated with caution, since mean shear rates depend on the corresponding reactor geometry and are not necessarily a good indication of the actual stress experienced by organisms in the fermenter. Measurement of a maximum shear rate and, thereby, a maximal stress, that floating particles can be subjected at the stirrer surface is complicated because of the close proximity to the rotating system. It is also subject to limitations in the temporal and spatial resolution capability of measurement systems available, such as particle image velocimetry (PIV). CFD simulations would necessitate extremely fine grids and small time steps, i.e., very expensive direct numerical simulation runs (DNS). The discrepancy between the principal knowledge about the types of particle-level collision events and shear stresses acting on the cells that occur and the integral computational quantities used for quantification appears large, accordingly.

For this reason, experimental substitute systems were developed with which isolated shear forces could be replicated and, specifically, isolated from the variety of possible force effects in a bioreactor. As a widely used model system for shear stress, the parallel plate flow chamber (abbreviation: PPFC) has become established in many areas of life sciences (Bakker et al., 2003; Bacabac et al., 2005). In a PPFC, nutrient medium flows between two parallel plates, overflowing the cells fixed on the lower plate surface. Due to the (usually) laminar character of the flow, parallel streamlines result. In the vicinity of the surface, a wall boundary layer is formed in which the fluid velocity drops to zero toward the immobile wall. The wall shear stress resulting from this gradient is largely homogeneously distributed in the PPFC, which results from the laminar flow in a shallow channel (Bakker et al., 2003). By mounting the PPFC on a microscope, the growth of microorganisms can be recorded over time and subsequently analyzed by image analysis. According to Brown (2000), the practical success of the PPFC is due to the homogeneity of the loading stimulus, the simplicity of the equipment, the straightforwardness of sampling, the interchangeability of the medium, and the simple access to the sample. However, the suitability of a PPFC as a reactor model system is considered poor because the turbulent flow phenomena in the reactor cannot be represented by equivalent laminar systems (Henzler, 2000).

With this shortcoming in mind, we extended the idea of experiments performed in a PPFC further in such a way that time- or location-variable shear stresses can be set within a single chamber device. The approach pursued in this work supplements the PPFC with a so-called backward-facing step in order to induce space- and time-dependent shear stresses in the step wake. The experimental setup and the CFD simulations are presented in Section 2, and first results and a discussion are given in Sections 3 and 4, respectively, while a brief introduction to the backward-facing step flow is introduced next.

### Flow Over a Backward-Facing Step

**Figure 1:**
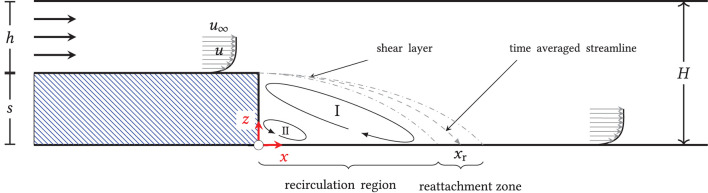
Principle of the backward-facing step flow; two-dimensional, schematic representation: when flow through a channel of height *h* is instantly expanded to height *H*, a shear layer develops downstream of the step. Due to the Kelvin-Helmholtz instability, vortices in that shear layer might form, build up, and induce turbulence, which then hits the region in the reattachment zone in the step wake. Upstream that zone a recirculation region develops, which includes a primary (I) and a secondary vortex (II).

The flow over a backward-facing step is a classical fluid-mechanical benchmark problem and has been investigated in numerous experimental and numerical studies (Eaton and Johnston, 1981; Armaly et al., 1983; Torczynski, 1992; Demuren and Wilson, 1994; Ratha and Sarkar, 2015; Toms, 2015). Figure 1 illustrates the basic flow phenomenon: fluid of constant density and viscosity flows inside of a closed channel with height *h*. After passing through a confined length of the channel, the chamber cross-section expands by the (step) height *s* to the total height *s* + *h* = *H*. Due to the instantaneous increase in cross section, the (wall) boundary layer arriving at the step detaches and forms a shear layer. Inside of it, unstable processes lead to the up-rolling and the transport of vortex structures in the wake, commonly referred to as Kelvin-Helmholtz instability (Rani et al., 2007). Behind the step, a low-pressure region with clockwise recirculating medium (I) develops, which leads to an entrainment of medium from the overlying shear flow, and, finally, to the reattachment of the flow at the bottom of the channel. Directly behind the step edge, a left-turning secondary vortex (II) may also form, depending on the particular flow regime.

As all processes are highly dynamic, the flow does not reattach at a specific location but rather in a reattachment zone with a time-averaged reattachment length *x*_r_. The mean and the instantaneous reattachment point can be characterized by a mean and instantaneous vanishing wall shear stress in the flow direction, respectively, i.e., τ_w, *i*_ = 0. For the presented, two-dimensional flow of an isotropic, incompressible fluid, τ_w, *i*_ is given by


(1) $$\begin{array}{l} \tau_{\text{w},i}\left(x\right)=\mu \frac{\partial u_{i}\left(x,z\right)}{\partial z}{|}_{z=0}. \end{array}$$

Here *u*_*i*_ denotes the velocity component in the *x*-direction, *z* the coordinate perpendicular to the wall, and μ the dynamic viscosity. A negative τ_w, *i*_ thus denotes backward flow, while a positive τ_w, *i*_ shows forward flow. At this point it is to be noted that real three-dimensional flows can also be characterized by the disappearance of the wall shear stress in the flow direction where the reattachment *line* *x*_r_(*x, y*) is two-dimensional, with *y* perpendicular to both *x* and *z*:


(2) $$\begin{array}{l} \tau_{\text{w},i}\left(x,y\right)=\mu \frac{\partial u_{i}\left(x,y,z\right)}{\partial z}{|}_{z=0}. \end{array}$$

Although numerous studies investigated the isolated effects of flow-specific (e.g., fluid velocity, degree of turbulence upstream of the step) and geometry-specific variables (e.g., expansion ratio *H*/*h*, chamber width, length of the upstream region), the combined effects under real conditions for a newly designed geometry, as done for this study, cannot be predicted sufficiently well. Therefore, the exact manifestation of the velocity field (and hence of the shear stress) must be either measured or inferred numerically.

Methods to measure wall shear stress range from pressure-based techniques (e.g., micro-electro-mechanical sensors, pressure-sensitive copolymers) to optical (e.g., particle image velocimetry) and chemical variants (e.g., electro diffusion; Böhm et al., 2014). For the small dimensions of the designed flow chamber (see below) this would be very challenging and was not tackled within this study. Instead, information about the fluid flow and the resulting wall shear stress inside of the backward-facing step channel was obtained numerically using a well-established software for computational fluid dynamics (CFD). This will be shortly explained in Section 2 after a description of the strain used, the experimental system, and the image analysis tools applied to evaluate microscopic time-lapse pictures.

## 2. Materials and Methods

### 2.1. Microbial Strain

In this study, the role of secretory vesicles (SV) was of interest. Secretory vesicles play a dual function inside the organism, as they transport cell wall-building enzymes (necessary for hyphal growth and cell wall stabilization) and products to the hyphal apex. For this, a strain was selected for which SVs and a chosen product, namely, glucoamylase, could be visualized with the help of a confocal microscope. The *A. niger* reporter strain MF31.2^1^ used in this study constitutively expresses a C-terminally dTomato tagged *glaA* gene for the target protein glucoamylase (GlaA) that is an alpha-glucosidase digestive enzyme. Moreover, the strain expresses an *egfp::sncA* sequence that labels the v-SNARE protein SncA attached to secretory vesicles, which are the primary transport vehicles for exocytosis and endocytosis in *A. niger*. Genotypic details of the genome modifications made in the MF31.2 strain and for the *egfp::sncA* sequence can be found in the work of Fiedler et al. (2018) and Kwon et al. (2014), respectively. The absorption spectrum of eGFP exhibits a maximum at 490 nm. In the experiment, excitation was performed with a blue laser of wavelength λ_blue_ = 488 nm, while detection of the emitted green light took place between 490–547 nm. The reporter protein dTomato has an absorption maximum at 540 nm and was excited with a green laser at λ_green_ = 552 nm. The emitted orange-red light was detected in a window of 651–716 nm.

### 2.2. Parallel Plate Flow Chamber With Backward-Facing Step

**Figure 2:**
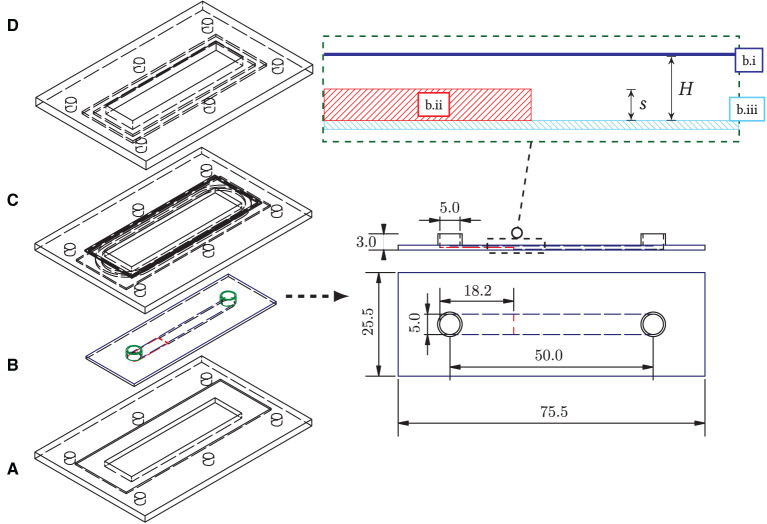
(Left) Overall structure of the backward-facing step flow chamber and the seal frame. **(A)** Bottom frame with recess for bonding (aluminum, milled); **(B)** Flow chamber (b.i: bottomless flow chamber, “Sticky slide 0.8,” *ibidi*; b.ii: step foil cutout, 400 μm, *Aslan*; b.iii: plastic cover glass, *ibidi*); **(C)** 3d-printed frame inlet with O-ring groove (ABS-like plastic “tough resin,” *Formlabs*); **(D)** Upper frame with countersink and groove (aluminum, milled). All components are connected by six nuts and bolts. An O-ring (54 × 1.5 mm, acrylonitrile butadiene rubber) distributes the pressure from above to the flow chamber. The chamber **(B)** is glued into the bottom frame **(A)** to prevent buckling of the plastic cover glass of the chamber due to the system pressure. (Right) Structure of the flow chamber with backward-facing step, dimensions in mm. Top: horizontal profile of the chamber assembly: the bottomless chamber (dark-blue) is glued into the cover glass slide (light-blue) with the step attached to it (red).

The prototype of the growth chamber was constructed out of a commercially available, bottomless, self-adhesive channel slide (sticky-Slide I Luer, catalog number 80, 168, *ibidi*) of total height *H* = 800 μm and width *b* = 5 mm that was glued onto a cover glass, which itself had the backward-facing step attached to it. For the backward-facing step, a polymer foil of height 400 μm was cut to the width of the channel recess, 5 mm, with a micro-cutter and completely enclosed by the channel slide after bonding. The bottomless channel was coated with an adhesive layer and was slipped over the composite of the polymer cover glass and the step inlet. The layered structure of cover glass, step, and attached channel slide is shown in Figure 2 on the right.

The step height *s* was set to half the total chamber height (400 μm), as most frequently used in the literature. For flow stabilization, a sufficiently long inflow zone upstream of the step was considered and was chosen as 27*s*.

### 2.3. Closed Circuit Pumping System

**Figure 3:**
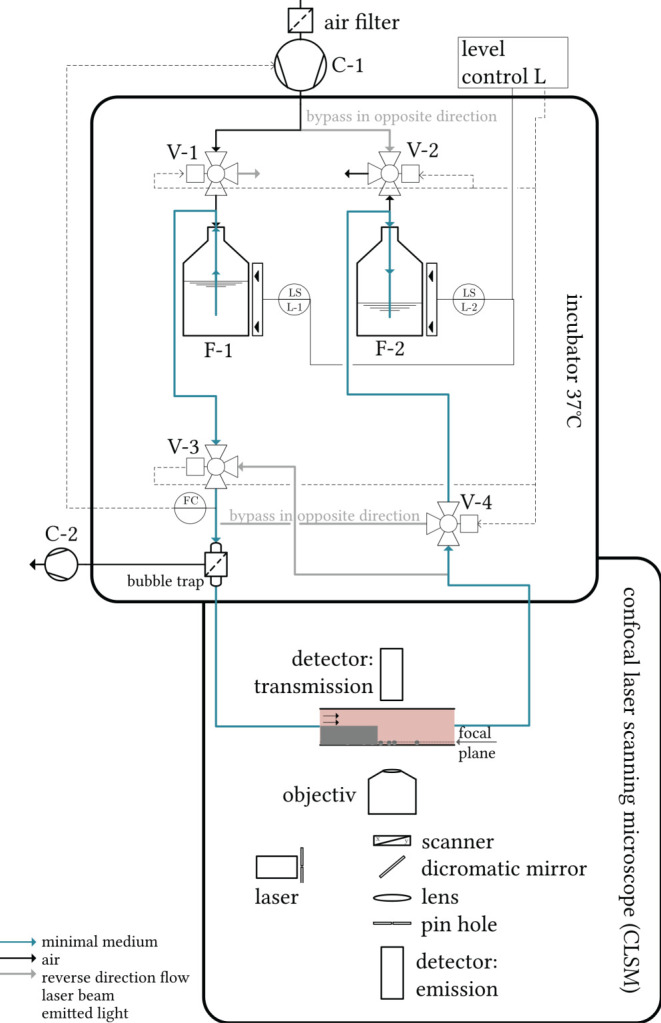
Schematic representation of the measurement setup. Tempered minimal medium is pumped alternately through the flow chamber (light-pink) *via* a compressor between two bottles. The flow chamber is mounted on a confocal laser scanning microscope, which focuses a laser beam on the sample probe. Both the laser transmission and the emitted light of excited fluorophores in the sample are detected in separate detector units in order to assess the local biomass and the local fluorophore density, respectively.

Experiments lasted for several hours, for which a continuous, uninterrupted flow of sterile medium over the backward facing step had to be applied. To guarantee this, a closed circuit pumping system was set up (see Figure 3). In an incubator, minimal medium (1 wt.-% glucose, according to Bennett and Lasure, 1991) was tempered to 37°*C* and pumped alternately back and forth between two glass bottles (F-1 and F-2, respectively, 250 mL, *Schott*). For an explanation of the functioning of the setup and for convenience, let us consider a situation in which bottle F-1, filled at first, while bottle F-2 is empty. Ambient air is drawn in through an air filter *via* the compressor(“AF-1,” *Elveflow*, *p*_max_ = 1, 400 mbar, C-1 in Figure 3) and compressed *via* valve V-1 into the space above the surface of the culture medium in F-1. The medium passes through valve V-3 and enters a bubble trap (“006BT-HF,” *Diba Industries*), in which a vacuum pump removes any air bubbles present from the liquid flow through a microporous, hydrophobic membrane. Venting is critical because hydrophobic fungal spores can agglomerate through interface contact, detach from the bottom of the coverslip, and be expelled from the flow chamber. The liquid then enters the flow chamber (light pink in Figure 3), where it overflows fungal samples adhering to glass surfaces. After passing through the microchannel, the medium reaches the bottle F-2 through the valve V-4, while the air from the top of the bottle expands from F-2 to the surroundings through the valve V-2.

The 2/3-way valves V-1 to V-4 switch as soon as the lower level sensor (capacitive level measurement, “XKC Y25 T12V,” *DFrobots*) in F-1 no longer detects any liquid. This releases the pressure in bottle F-1 to the surroundings (valve V-1 switches) and builds up the pressure in bottle F-2. The fluid is directed through valve V-4 into a recirculating tube and reaches the flow cell in an unchanged flow direction (in Figure 3 from left to right). The entire experimental setup thus ensures a pumping process without the need to replace or refill nutrient medium. The sterility of the compressed air and medium is ensured by the filters in the suction port. Rigid PFA tubing (“TYGON”®0.5 × 1.6 mm, *Saint-Gobain*) was chosen on the air side of the pumping system, while silicone tubing (“Versilon”®SPX-50 2.4 × 5.6 mm, *Saint-Gobain*) was used on the water side. In this system, the volume flow (measuring point FC, Coriolis-based flow sensor, “mini CORI-FLOW,” *Bronkhorst*) was controlled by the system pressure above the liquid. The controller used was a simple PI controller, which was manually adjusted *via* the compressor manufacturer's software “Elveflow smart interface” for the volume flow rates studied (see Section 2.4). A preheating time of 3h before starting the measurements guaranteed the uniform temperature control of the microscope, medium, tubes, and flow chambers.

### 2.4. Experimental Details

The flow chamber was mounted on a confocal laser scanning microscope (”SP8,” *Leica*) which focused a laser beam on the sample probe. Both the laser transmission and the emitted light of excited fluorophores in the sample were detected in separate detector units in order to assess the local biomass and the local fluorophore density, respectively. The confocal laser scanning microscope was further enclosed in a plexiglas box into which tempered air was fed.

**Figure 4:**
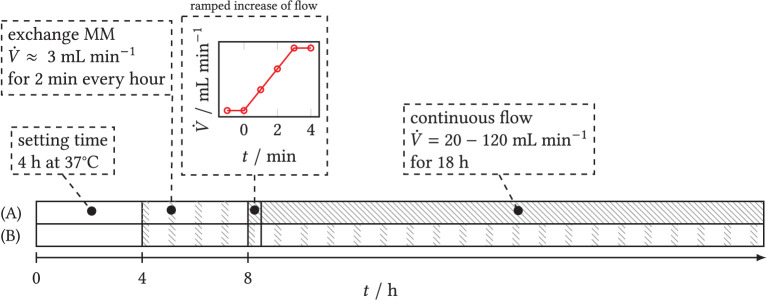
Time schedule of flow experiments with step chamber. Gray shaded: active flow. **(A)** Experiments with target volume flow. After a settling time of 4 h, the step chamber is connected to the pumping system and flowed through at intervals of 2 min h^−1^ each time over 4 h to ensure sufficient oxygen saturation. A ramp is used to set the target flow rate of the main experiment and maintain it over 18 h. **(B)** Blank sample: intermittent exchange of medium starting at *t* ≥ 4 h over the entire duration of the experiment.

The time course of the flow experiments is plotted as a time line in Figure 4. The prepared backward-facing step channel was coated with a Poly-L-Lysine solution to allow for spore adherence to the surface behind the step. It was then filled with spore solution and stored in the incubator at 37°*C* for the first 4 h to allow the spores to sink to the bottom of the chamber and to adhere. Then, the step chamber filled with spore solution was connected to the pumping system. To avoid a lack of dissolved oxygen in the solution, minimal medium was subsequently exchanged every full hour for 2 min each time, i.e., at intervals with a low volume flow of 3 mL min^−1^. The target volumetric flow rate of the experiment was set after 8 h in a ramped trajectory over 3 min to avoid high temporal pressure and shear gradients. During the main experiment, a quasi-continuous flow was maintained inside the closed circuit (see Section 2.3) for at least 18h. Four flow experiments were performed in which nutrient medium was pumped through the step chamber at $\dot{V}=\left\{20,50,80,120\right\}\;\text{mL}\;{\text{min}}^{-1}$. During the experiments, microscopic time-series images were taken in three spatial dimensions to record the changes in biomass concentration and fluorescence of the secretory vesicles and of GlaA downstream of the step over time. In the following CFD simulations, 150 mL min^−1^ was considered as well. However, in the experiments, this led to an increased outwash of spores.

**Figure 5:**
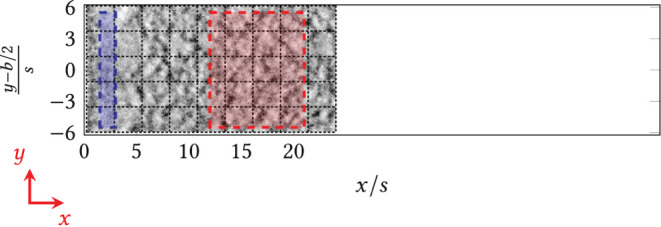
Raster images of the area behind the step (*x* = 0) taken at the confocal laser-scanning microscope, i.e., individual images were merged after the experiments (function tile merge). Blue/red: Designated areas for the quantification of biomass and fluorescence. Blue: area directly behind the step; red: area downstream of the step/the reattachment zone.

For four volume flows $\dot{V}=\left\{20,50,80,120\right\}\;\text{mL}\;{\text{min}}^{-1}$, raster overview images were taken according to Figure 5, as rectangles with broken *black* lines, consisting of individual image data stacks in three spatial dimensions over time and in three channels (light transmission and fluorescence channels (green eGFP and red dTomato)). The acquisition settings on the confocal laser scanning microscope are deposited in the Supplementary Table 1. The individual image data stacks were merged into one overall image in the LASX acquisition software (*Leica*, function: tile merge).

### 2.5. Image Analysis

Light absorption and fluorescence emission of the raster images (see Section 2.4) were quantified using scripts in the open-source program FIJI (Schindelin et al., 2012). For readers unfamiliar with the particular filter names in FIJI, the steps in image analysis are laid out below as mathematical operations. For an image stack in three spatial, and one time dimension, thus, consisting of M pixels in *x*, N pixels in *y*, O pixels in *z* direction and *N*_*t*_ time frames with


(3) $$\begin{array}{l} \begin{array}{l} \left\{\text{m}\in \mathbb{N}\;|\;1\leq \text{m}\leq \text{M}\right\}\\ \left\{\text{n}\in \mathbb{N}\;|\;1\leq \text{n}\leq \text{N}\right\},\\ \left\{\text{o}\in \mathbb{N}\;|\;1\leq \text{o}\leq \text{O}\right\},\\ \text{and}\\ \left\{t_{i}\in \mathbb{R}\;|\;t_{1}\leq {\text{t}}_{i}\leq t_{N_{t}},\;i=1,2,\ldots N_{t}\right\} \end{array} \end{array}$$

a dataset of the form I[m, n, o, *t*_*i*_] can be constructed. Three-dimensional (+ time) image data sets were obtained in three channels, namely, transmission (T), green and red fluorescence (T). The following programs quantify light extinction by the biomass in the transmission channel, T^−1^, and the *z*-sum of the axial fluorescence channels, $T_{\underset{z}{\sum }}$.

#### Biomass

The change of biomass concentration can be calculated from how much light the sample absorbs. Due to the power conservatism of the transmission radiation, an arbitrary height plane of the sample image can be chosen to determine the amount of overall biomass (Zhang et al., 2007). The middle planes o^*^ = (O−1)/2 of all *N*_*t*_ frames were inverted to obtain the intensity of absorbed light (extinction) as positive intensity values:


(4) $$\begin{array}{l} T{\;}^{-1}\left[\text{m},\text{n},{\text{o}}^{*},t_{i}\right]=N_{\text{gray}}-1-T\left[\text{m},\text{n},{\text{o}}^{*},t_{i}\right]\;, \end{array}$$

with the number of gray values of the digital image, *N*_gray_. The spatial **local mean** in an area of the size M × N pixels of the time sequence is calculated by


(5) $$\begin{array}{l} \langle T{\;}^{-1}\left[{\text{o}}^{*},t_{i}\right]\rangle =\frac{1}{\text{M }\cdot \text{ N}}\overset{\text{M}}{\underset{\text{m}=1}{\sum }}\overset{\text{N}}{\underset{\text{n}=1}{\sum }}T{\;}^{-1}\left[\text{m},\text{n},{\text{o}}^{*},t_{i}\right]. \end{array}$$

The variance is the sum of the square difference from the local mean, and can, therefore, be obtained by


(6) $$\begin{array}{l} \langle \langle T^{-1}\left[{\text{o}}^{*},t_{i}\right]\rangle \rangle =\frac{1}{\text{M }\cdot \text{ N}}\overset{\text{M}}{\underset{\text{m}=1}{\sum }}\overset{\text{N}}{\underset{\text{n}=1}{\sum }}{\left(T^{-1}\left[\text{m},\text{n},{\text{o}}^{*},t_{i}\right]-\langle T{\;}^{-1}\left[{\text{o}}^{*},t_{i}\right]\rangle \right)}^{2}. \end{array}$$

#### Total Axial Fluorescence

The laser scanning microscope allows for the layer-by-layer resolution of fluorescence in the sample. The **total axial fluorescence** over all O slices is calculated for all pixels in all *N*_*t*_ frames:


(7) $$\begin{array}{l} F_{\sum_{z}}\left[\text{m},\text{n},t_{i}\right]=\overset{\text{O}}{\underset{\text{o}=1}{\sum }}F\left[\text{m},\text{n},\text{o},t_{i}\right]. \end{array}$$

The spatial local mean follows by subsequent averaging over [m, n]


(8) $$\begin{array}{l} \langle F_{\sum_{z}}\left[t_{i}\right]\rangle =\frac{1}{\text{M }\cdot \text{ N}}\overset{\text{M}}{\underset{\text{m}=1}{\sum }}\overset{\text{N}}{\underset{\text{n}=1}{\sum }}F_{\sum_{z}}\left[\text{m},\text{n},t_{i}\right] \end{array}$$

and gives the time-dependent, (*z*)-summed, averaged fluorescence per area.

### 2.6. CFD Simulation Details

The expected wall shear stress in the backward-facing step chamber was estimated by dynamical fluid simulations in a commercially available software environment (StarCCM+, *Siemens*). The geometrical negative of the internal channel volume was discretized into 9.63·10^6^ hexahedral cells, which were condensed around the step to ensure sufficient resolution in the step vicinity. The turbulence in the step wake was captured using a large eddy vortex model (LES) that resolves bigger structures in the interior of the flow, whereas smaller near-wall vortices were described with a simpler Reynolds-Averaged Navier-Stokes (RANS) approach. For further details of the discretization, the reader is referred to Supplementary Figures 1–3.

## 3. Results

### 3.1. Computational Fluid Dynamics: Wall Shear Stress

**Figure 6:**
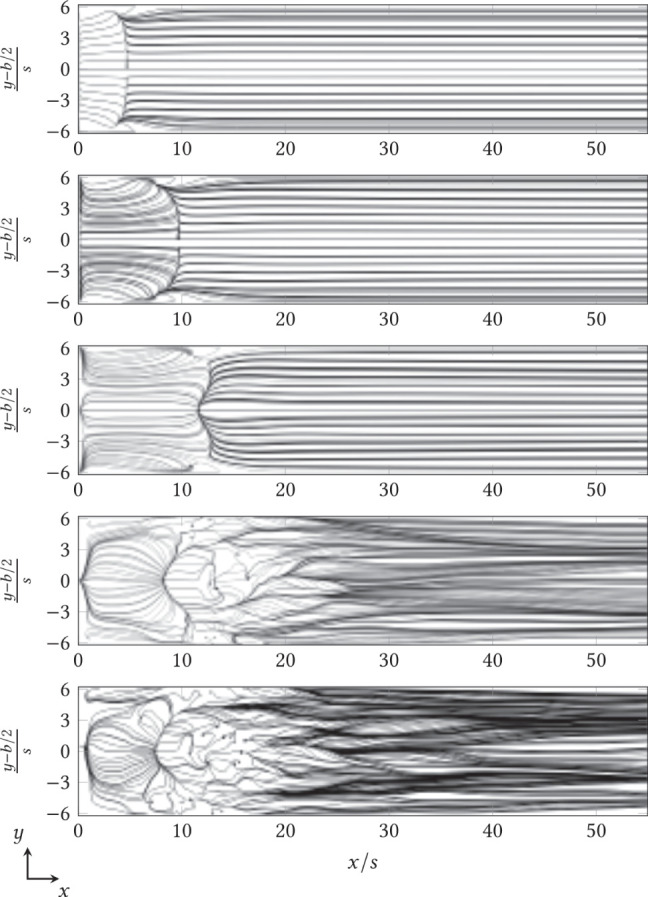
Instantaneous progression of streamlines above the surface of the lower cover glass behind the backward-facing step (at *x*/*s* = 0) for $\dot{V}=\left\{20,50,80,120,150\right\}\;\text{mL}\;{\text{min}}^{-1}$ (top to bottom) in plan view. The channel width (shown here as *y*-axis) was also normalized to the step height *s* and centered, according to convention.

Figure 6 shows the instantaneous streamlines constrained to the top of the lower coverslip of the step chamber at arbitrary times for $\dot{V}=\left\{20,50,80,120,150\right\}\;\text{mL}\;{\text{min}}^{-1}$ in plan view. In this plot, the medium fluxes in the immediate vicinity of the adherent fungus are visible. For these, the constrained streamline function in StarCCM+ was used to integrate the velocity field near the bottom and project the resulting path in the plane. Starting from 375 seeding points of the streamlines (*black points* each in Figure 6), each was integrated forward and backward in time. For $\dot{V}=\left\{20,50,80\right\}\;\text{mL}\;{\text{min}}^{-1}$, it is clear that the flow was in orderly parallel paths in the laminar regime. The primary vortex guided the streamlines back to the step edge in overlap-free paths, while the flow behind the primary vortex quickly touched down at the bottom of the chamber and left the component in an equally ordered fashion. From $\dot{V}=120\;\text{mL}\;{\text{min}}^{-1}$, local vortices formed behind the reattachment line, extending at $\dot{V}=150\;\text{mL}\;{\text{min}}^{-1}$ over the near-wall regions into the primary vortex behind the backward-facing step. Laminar, uniform profile flow was not achieved in the transient region, so streamlines crossed even after reattachment.

**Figure 7:**
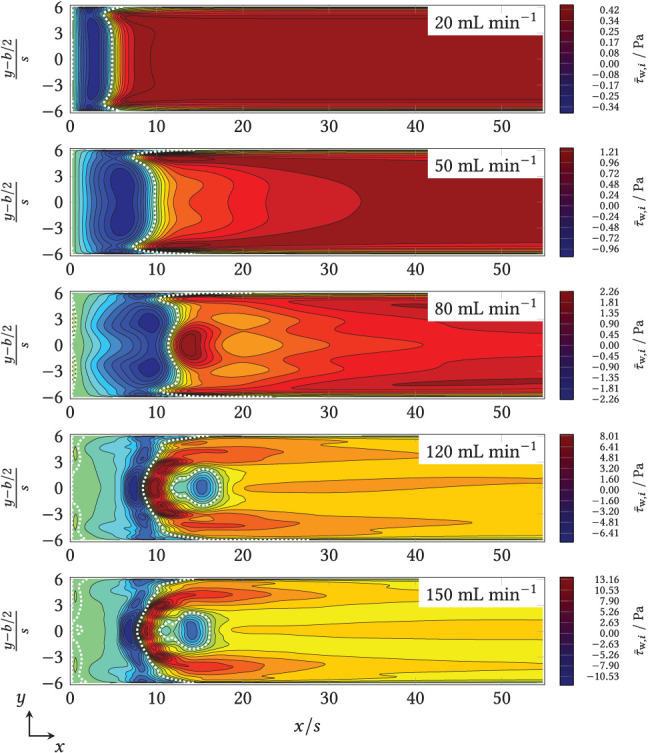
*x*-component of the time-averaged wall shear stress (τ_w, *i*_) in the zone downstream of the step (*x*/*s* = 0) for $\dot{V}=\left\{20,50,80,120,150\right\}\;\text{mL}\;{\text{min}}^{-1}$ (from top to bottom). The channel width (shown here as *y*-axis) was also normalized to the step height, *s*, and centered, according to convention.

The distribution of the time average of the *x* component of the wall shear stress, ${\bar{\tau }}_{\text{w},i}\left(x,y\right)$, and the time variance, ${\bar{\bar{\tau }}}_{\text{w},i}\left(x,y\right)$, on the bottom of the flow chamber were obtained by averaging the computed velocity fields over *N*_*t*_ simulated time steps. For the full field plots of the mean and the variance of the wall shear stress magnitude, the first and the second vector component, the reader is referred to Supplementary Figures 6–15. The field data for the first vector component of the mean wall shear stress, ${\bar{\tau }}_{\text{w},i}\left(x,y\right)$, is compared in Figure 7 as contour plots for all volume flows investigated. The mean reattachment line with ${\bar{\tau }}_{\text{w},i}\left(x,y\right)=0$ is visible here as a white dotted line. From Figure 7, different trends for the distribution of the time-averaged *x*-component of the wall shear stress–and thus the reattachment line–can be seen. The length of the negative-valued ${\bar{\tau }}_{\text{w},i}\left(x,y\right)$ region increases until $\dot{V}=80\;\text{mL}\;{\text{min}}^{-1}$ and is then pushed back upstream. From $\dot{V}=80\;\text{mL}\;{\text{min}}^{-1}$, a positive-valued maximum of τ_w, *i*_(*x, y*) forms behind the mean reattachment length. From 120 to 150 mL min^−1^, a midpoint reverse flow region also forms at 10 ≤ *x*/*s* ≤ 17. According to Armaly et al. (1983), who mentioned this region for the first time, this is typical of the incipient transient flow regime.

The reattachment line also depends on the distance to the side walls of the chamber: In the laminar regime, the primary vortex is shortened upstream near the sidewalls, whereas in the transient regime, the line of restart is pulled or appears to blur downstream near the sides. The former phenomenon occurred because the sidewalls slowed down the core flow. The latter was caused by several overlapping effects, including the increased velocity of the steady-state boundary values in the inlet (see Supplementary Figure 4).

From the time-averaged results of simulated volume flows, $\dot{V}=\left\{20,50,80,120\right\}$ mL min^−1^, the mean and the maximal wall shear rates were calculated, resulting in $\bar{\dot{\gamma }}=\left\{694,1,736,2,774,4,166\right\}$ s^−1^ and in ${\dot{\gamma }}_{\text{max}}=\left\{694,1,953,3,804,13,019\right\}$ s^−1^, respectively. Whereas the expected $\bar{\dot{\gamma }}$ exceed the (above-cited) reference range of 1−1, 000 s^−1^ from fermenters for $\dot{V}\geq 50\;\text{mL}\;{\text{min}}^{-1}$, the authors did not find respective data for maximal local shear rates for fermenters in literature.

### 3.2. Macroscale Growth Patterns

**Figure 8:**
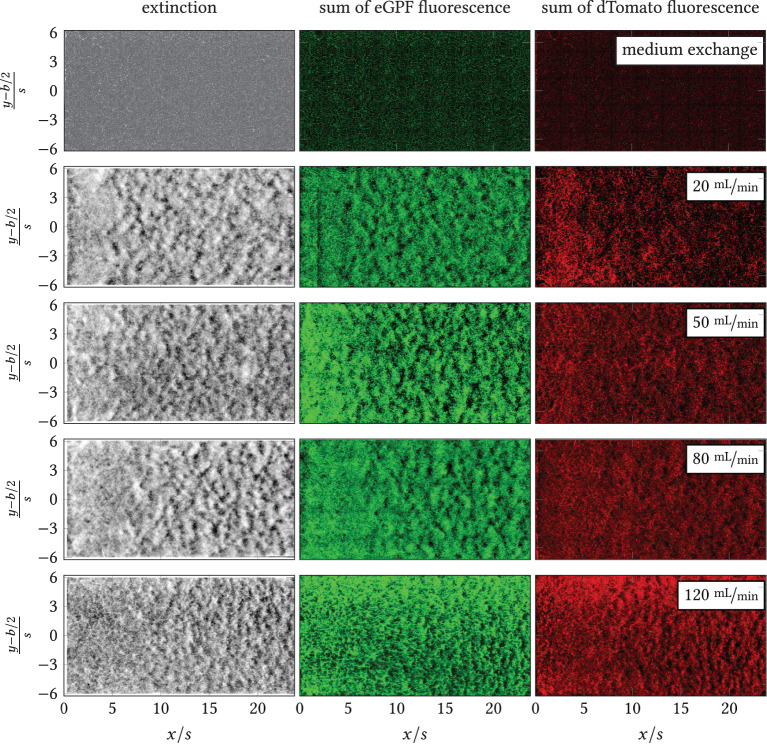
Overview images of extinction, sum of eGFP fluorescence and dTomato fluorescence downstream of the step (left image margin, *x*/*s* = 0) for the blank sample (exchange of minimal medium on minimal shear) and $\dot{V}=\left\{20,50,80,120\right\}\;\text{mL}\;{\text{min}}^{-1}$ . All pictured distributions were measured at the last time point per experiment, i.e., *t*_end_ = {26.13, 23.48, 22.50, 24.10, 22.42} h. *Black* line: time-averaged reattachment line *x*_r_(*x, y*) per $\dot{V}$. The color space of each image has been adjusted for the highest possible contrast. Intensity-based differences between images are, thus, not equivalent to quantitative differences.

In the developed backward-facing step flow chamber (see Figure 2) operated in the pumping system (Figure 3) a series of experiments of increasing volume flows $\dot{V}=\left\{20,50,80,120\right\}$ mL min as well as for a blind sample with low-shear medium flow were conducted. The resulting overview images behind the backward-facing step are shown in Figure 8 for the extinction and the total green and red fluorescence. Thus, dark regions indicate a low biomass or fluorophore density, while bright regions stand for a high density. The reattachment lines as estimated by the CFD simulations are plotted onto the extinction images as well.

**Figure 9:**
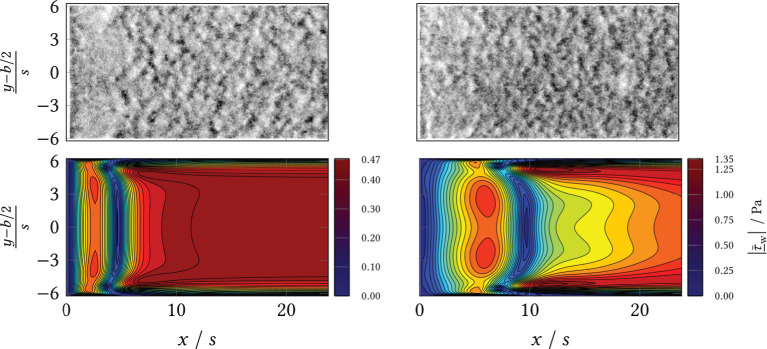
**(Top)** Wide field microscopy measurements in the area behind the BFS (*x*/*s* = 0) and separation line (*black*). **(Bottom)** Simulation results (CFD) of the mean shear stress magnitude. Left: volumetric flow rate $\dot{V}=20\;\text{mL}\;{\text{min}}^{-1}$; Right: volumetric flow rate $\dot{V}=50\;\text{mL}\;{\text{min}}^{-1}$.

**Figure 10:**
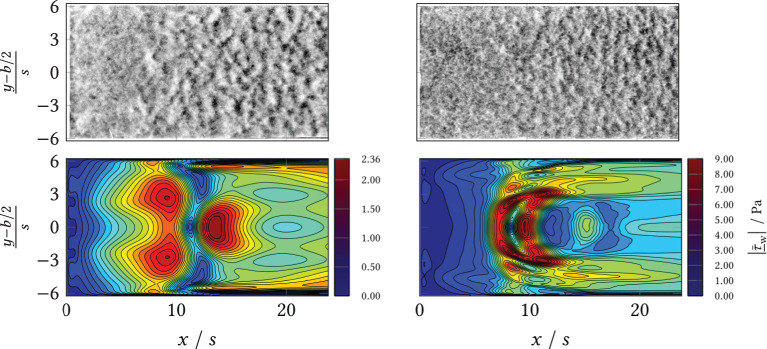
**(Top)** Wide field microscopy measurements in the area behind the BFS (*x*/*s* = 0) and separation line (*black*). **(Bottom)** Simulation results (CFD) of the mean shear stress magnitude. Left: volumetric flow rate $\dot{V}=80\;\text{mL}\;{\text{min}}^{-1}$; Right: volumetric flow rate $\dot{V}=120\;\text{mL}\;{\text{min}}^{-1}$.

During the experiments, different macromorphological structures formed in the step wake. These can be seen in the extinction and the eGFP and dTomato fluorescence overview images, Figure 8: the zone directly behind the step was homogeneously overgrown, i.e., the mycelia density appeared uniform. Further downstream, the mycelia structure changed visibly. The structure appears “flocculent,” i.e., coherent areas of higher and lower extinction or fluorescence were formed, which extended to the channel sides. These yielded an almost regular-looking metastructure in which light and dark zones alternated. The transition between the two zones appeared smooth and coincided roughly with the position of the predicted time-mean reattachment line *x*_r_(*x, y*) (*black* in Figure 8). In particular, for 20 mL min^−1^, the sharply defined boundary between the zones coincides exactly with the time-averaged reattachment line *x*_r_(*x, y*). For the higher volume flows, the observed boundary in the sample became softer, which, in turn, could be associated on the one hand with increased variability of the actually time-dependent *x*_r_(*x, y, t*) with increasing flow. On the other hand, the more flocculent structures appear in areas of higher absolute shear stress $\left|{\underline{\bar{\tau }}}_{w}\right|$ as shown by a comparison of Figure 8 with Supplementary Figures 6, 8, 10, 12 from the Supplement. To highlight this, Figures 9, 10 contrast the same cutouts of Figure 8 and Supplementary Figures 6, 8, 10, 12 for all volume flows. Although for *x*/*s*≈10 the mean absolute shear stress is low for 80 mL min^−1^, Supplementary Figure 9 predicts a high variability in that region, i.e., the hyphae experience significant shear of varying sign. For 120 mL min^−1^, the nature of the structures changed as they became finer and more cross-linked in both zones and the area of individual flakes decreased. Here, the shear stresses are higher as well in the anterior region compared to lower flow cases (see Supplementary Figure 12). All described structural changes became apparent in the images at about 13 h after the start of the continuous flow through the channel.

#### 3.2.1. Biomass

**Figure 11:**
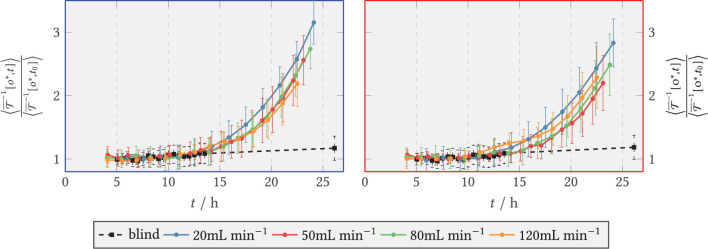
Relative light extinction for $\dot{V}=20,50,80,120\;\text{mL}\;{\text{min}}^{-1}$ and the blank sample (shear-free medium exchange). The light extinction was normalized to the initial extinction at *t*_0_ = 9 h. The error bars bound the 1σ interval around the zone mean and quantify the scatter of the extinction per zone. **(Left)** Zone behind the step (recirculation zone); **(Right)** Zone downstream of the step, *x*/*s* > 15 (after reattachment).

The extinction of light by the biomass was determined *via* the transmission channel and a time course of the relative biomass concentration was calculated. These are relative values, as they were divided by the extinction value at the reference time *t*_0_ = 9 h. This time point seemed reasonable because in previous experiments with a similar flow sequence and with a higher magnification, to allow for an observation of individual spores (data not shown) only a neglectable amount of spores/germlings were flushed out later than 8 h from the start of the experiment. Thus, normalization was done with respect to the remaining biomass. The calculated biomass plots can be found in Figure 11 for the front area directly behind the step, see blue area in Figure 5, and for the back zone downstream (red area) for all $\dot{V}$ (plus a blank sample, containing spores without slow interval medium exchange) as regional mean values. The standard deviation–identifiable as an error bar in the figure–describes the dispersion of extinction per zone (i.e., directly behind or downstream of the step) and is thus indicative of the distribution of biomass per area^2^.

It can be seen that the smallest increase in biomass (up to 1.2 times the reference) was obtained in the blank sample. It should be noted that this was the final measured value, as no images were available for the blank sample between *t* = 13 h and *t* = 26 h due to an error in the microscopy operation. For continuous shear flow, i.e., for $\dot{V}=\left\{20,50,80,120\right\}\;\text{mL}\;{\text{min}}^{-1}$ , accelerated growth was present initially, followed by smaller, near linear growth of biomass. Figure 11 covers only the onset of this transition, as the experiments were aborted after around 24 h. The increase pattern was similar for all volume flows, but occurred with a time lag. Here, the mean extinction in the anterior zone was slightly higher than in the posterior zone. Overall, the comparison of the two zones shows that the time courses of light extinction by biomass at the different volume flows were very similar to each other. This is especially true when the wide scatter of the absorbance per zone is taken into account. The scatter width resulted from the inhomogeneous distribution of the biomass. After *t* = 8 h, a small decrease in biomass is noticed before the higher volume flows and especially in the posterior zone. This resulted from the detachment of spores by the flow and was accounted for by normalizing to the reference time *t*_0_ = 9 h.

#### 3.2.2. Secretory Vesicles (SV-eGFP)

**Figure 12:**
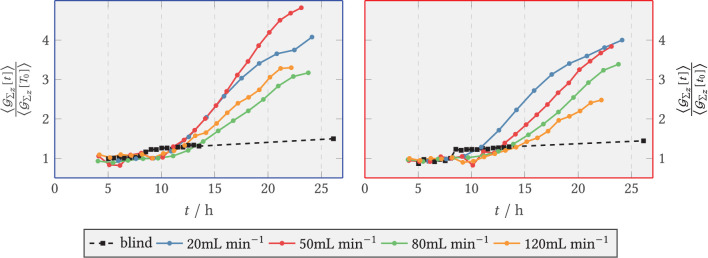
Relative axial green fluorescence (G) sum of tagged secretory vesicles for $\dot{V}=20,50,80,120\;\text{mL}\;{\text{min}}^{-1}$ and the blank sample (shear-free medium exchange). The fluorescence was normalized to the initial extinction at *t*_0_ = 9 h. **(Left)** Zone behind the step (recirculation zone); **(Right)** Zone downstream of the step, *x*/*s* > 15 (after reattachment).

The quantitative course of the relative fluorescence sum in the zone directly behind the step and in the downstream area is depicted in Figure 12, *left* and *right*, respectively. During the first hours of the experiments the amount of vesicles formed increased exponentially for all $\dot{V}$, quickly transitioned to approximately linear growth, and leveled off from *t* = 19 h on in the front region and for *t* = 17 h in the back region, respectively. The blank sample did not show exponential growth behavior and resembled a flat linear progression. From 8 h, the relative green fluorescence of the blank sample increased briefly in both zones and remained at about the same level until the end of the experiment. During the period from 8–10 h, the green fluorescence in the experiments with medium flux dropped temporarily because fluorescent spores were flushed out of the system. For compensation, in each case, the entire time series was normalized to the spore fraction still present at 9 h. The total sum of fluorescence was visibly higher in the anterior zone than in the posterior zone at $\dot{V}=\left\{50,120\right\}\;\text{mL}\;{\text{min}}^{-1}$. The fluorescence sum of SV increased rapidly in the anterior zone for 20 and 50 mL min^−1^, whereas at the higher volume flows of 80 and 120 mL min^−1^ the increase was delayed and with a lower slope. The final fluorescence sum achieved was highest at 50 mL min^−1^ with 4.8-fold fluorescence sum, while 80 and 120 mL min^−1^ had a maximum of 3.1-fold vesicle activity.

In the downstream zone, the exponential increase graded according to the volume flow, i.e., 20 mL min^−1^ being the first and 120 mL min^−1^ the last. The maximum slope of the relative fluorescence sum here also decreased according to the volume flow rate, so that the highest maximum slope was reached for 20 mL min^−1^ and the lowest for 120 mL min^−1^. For all cases, the curves flattened out from *t* ≥ 21 h at the latest.

#### 3.2.3. Glucoamylase (GlaA-dTomato)

**Figure 13:**
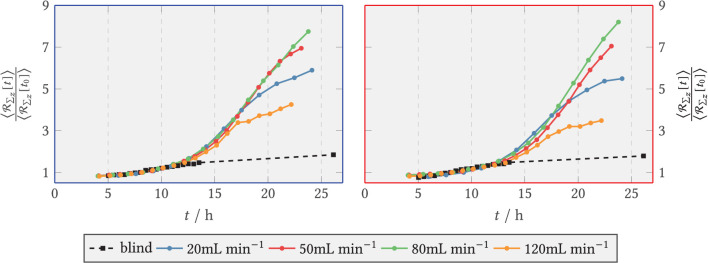
Relative axial red fluorescence (R) sum of tagged glucoamylase for $\dot{V}=20,50,80,120mLmin^{-1}$ and the blank sample (shear-free medium exchange). The light extinction was normalized to the initial extinction at *t*_0_ = 9 h. **(Left)** ZONE behind the step (recirculation zone); **(Right)** Zone downstream of the step, *x*/*s* > 15 (after reattachment).

**Figure 14:**
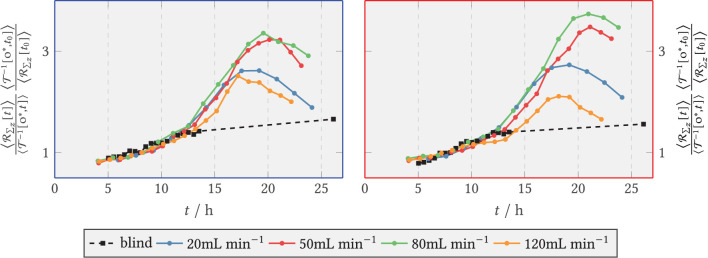
Relative axial red fluorescence (R) sum of tagged glucoamylase for $\dot{V}=20,50,80,120\;\text{mL}\;{\text{min}}^{-1}$ and the blank sample (shear-free medium exchange) divided by the relative biomass per time. The light extinction and the biomass were normalized to the initial extinction and the initial biomass at *t*_0_ = 9 h. **(Left)** Zone behind the step (recirculation zone); **(Right)** zone downstream of the step, *x*/*s* > 15 (after reattachment).

Analogous to green fluorescence, red fluorescence emitted by the marker protein dTomato at the target enzyme GlaA was evaluated. The results are shown in Figure 13. The zone-specific courses of total fluorescence of dTomato-labeled GlaA are plotted as relative values for the blank sample and for all volume flows. The fluorescence sum took relatively similar exponential initial courses for the volume flows studied. After an exponential rising phase, the activity in both zones flattened out after 17 h for all volume flows. For both zones, significantly less GlaA was formed at 120 mL min^−1^ than at the remaining volume flows. As can be seen in the *right* part of Figure 13, the activity in the posterior zone at 120 mL min^−1^ was lower than in the anterior zone. In contrast, GlaA at 80 mL min^−1^ increased longer in the posterior zone compared with the anterior one, reaching a maximum value of 8 times the relative fluorescence sum. At the other volumetric flows studied, there were only relatively small differences in GlaA activity between the anterior and posterior zones. Figure 14 displays the relative GlaA amount divided by the relative biomass. A lower relative GlaA production was obtained both for low and high flow rates while intermediate flow rates gave rise to higher values.

## 4. Discussion

While there was little difference in the mean relative amount of biomass, the formed mycelium showed designated areas of varying metastructures in all flow-through experiments. The boundary line between the two zones roughly coincided with the course of the time-mean reattachment line *x*_r_(*x, y*) that was predicted by the preceding fluid simulation. The mycelia in the low absolute shear zone directly behind the step, where concomitantly the shear stress varied only slightly over time, were characterized by homogeneous and uniform structures. In contrast, structures were inhomogeneous, cloud-like more downstream of the step in regions of higher absolute shear stress or regions with low mean absolute shear stress but with a high variability. The latter occurred in the reattachment region, where the flow dynamically switches between downward and backward flow and thereby dynamically impacts on the mycelial structures. It should be noted that the proportion of *y*-directed flow, i.e., orthogonal to the main flow, was significantly higher in the anterior zone than downstream of *x*_r_(*x, y*) and grows with increasing flow velocity (compare three dimensionality of the flow in Supplementary Figure 5). However, the extent to which this flow component could favor uniform hyphal growth remains an open question.

These observations clearly show that the absolute shear stress and its variation have a distinctive influence on the macromorphology. While this correlation had to be expected, the experiments in the newly designed flow chamber allow for a first step toward a quantitative description. Depending on the volume flow chosen, more or less pronounced vortex structures can be produced in the flow behind the backward-facing step. These vortical structures hit at the bottom of the chamber downstream of the step producing shear stress and variations of shear stress that can be manipulated by the volume flow. Observing germinating spores in that region microscopically then more clearly correlates mechanical stimulus with developing morphology of mycelia, especially when compared to experiments run in a reactor for which only integral quantities can be given. However, in this study, larger regions of the flow chamber were assessed microscopically with a smaller magnification to determine statistically more sound estimates of various quantities. This magnification did not allow for a finer characterization of the (micro-)morphology. Such investigations have to be performed in a following study.

This work started from a hypothesis that for cultivations in a higher shear stress environment more secretory vesicles (SV) are needed to transport cell wall-building components to the tip and contribute to thicker cell walls to withstand the mechanical stress. If the amount of SVs is limited, this would mean that less SVs are available for product secretion. The amount of SVs present was quantified by the *z*-sum of the fluorescence signal emitted by the protein eGFP in the *A. niger* mutant used, which labeled the SVs. As the SNARE-proteins are attached to the vesicle surface, the signal is related to the vesicle surface area. Assuming SNARE-density on the vesicle surface to be constant, the signal is regarded as proportional to the number of vesicle instances. The relative progress indicated an accelerated increase in secretory vesicles at the beginning of cultivation, which flattened slightly after 20 h concomitantly with near linear biomass growth. The secretory vesicle density decreased overall when the volume flow rate increased. This behavior was most evident in the posterior zone, where differences in predicted averaged wall shear stress between volume flows were also more pronounced. The secretory vesicle density per biomass (data not depicted in this study) also decreased from $\dot{V}=80\;\text{mL}\;{\text{min}}^{-1}$. Considering the double role of secretory vesicles according to which secretory vesicles are equally used for the transport of cell wall-stabilizing components and of secreted products, this tendency seems surprising. In this regard, it contradicts the assumption that *A. niger* would, in case of increased hydrodynamic stress, enlarge the amount of secretory vesicles for the purpose of cell wall stabilization. Whether subapical cell wall strengthening with a different dynamical role of SVs takes places in such instances is not known.

Additionally, it cannot be completely ruled out that the secretory vesicle density was lowered with increasing volume flow due to a flush-out of spores from the corresponding zones. Respective investigations of the adhesion of spores in long-term tests are to be carried out in the future.

As a product to be secreted by SVs, the progression of GlaA was measured by summing the dTomato red fluorescence. Here, the finding published by Hayakawa et al. (2011) and Fiedler et al. (2018) that GlaA accumulates in the septa of the fungus was initially confirmed. As in the time course of GlaA formation, the initial exponential increase in GlaA (as in secretory vesicles) transitioned to a linear increase as seen for the biomass as well. However, the volume fluxes studied were not significantly different from each other for $\dot{V}=\left\{20,50,80\right\}\;\text{mL}\;{\text{min}}^{-1}$ and similar relative changes were obtained in the anterior and posterior zones. For the blank sample, the least GlaA was produced. Among the flow-through samples, 120 mL min^−1^ showed the lowest GlaA density, with the slope curve flattening visibly after 17 h. Evaluating the GlaA density divided by the biomass density suggests that a sufficiently high volumetric flow rate can significantly increase the yield of GlaA, but too strong a loading stimulus tends to decrease the production rate per biomass. A correlation with the amount of secretory vesicles cannot be ruled out here, since secretory vesicles transport GlaA to the cell membrane. A decreased number of secretory vesicles present could thus prevent or slow down the secretion of GlaA. Although vesicle fluorescence decreased with increasing flow rate, GlaA fluorescence remained at least at similar levels up to and including 80 mL min^−1^ and thus apparently unaffected by the reduction of secretory vesicles. However, an influence of secretory vesicles and GlaA on the formation of new biomass could not be detected. Still, as the lowest GlaA amount was observed for the highest mechanical stress, the initial hypothesis could not be ruled out with this finding.

In assessing the influence of flow on the fungus, other fluid mechanics criteria should additionally be used, considering, for example, energy dissipation during vortex decay behind the backward-facing step and vortex size-specific influence on hyphae, which all have to be postponed to future studies.

## Data Availability Statement

The raw data supporting the conclusions of this article will be made available by the authors, without undue reservation.

## Author Contributions

PK contributed to conception and design of the study, performed the experiments, the statistical analysis, the simulations, and wrote the first draft of the manuscript. RK corrected and wrote sections of the manuscript. All authors contributed to manuscript revision, read, and approved the submitted version.

## Funding

The authors thank the Deutsche Forschungsgemeinschaft (DFG) for the financial support for this work (KI 679/10-1) within the SPP 1934 DiSPBiotech.

## Conflict of Interest

The authors declare that the research was conducted in the absence of any commercial or financial relationships that could be construed as a potential conflict of interest.

## Publisher's Note

All claims expressed in this article are solely those of the authors and do not necessarily represent those of their affiliated organizations, or those of the publisher, the editors and the reviewers. Any product that may be evaluated in this article, or claim that may be made by its manufacturer, is not guaranteed or endorsed by the publisher.

## Footnotes

^1^Nomenclature of the mutant strain according to the internal master database of the chair of Applied Molecular Microbiology at TU Berlin, Germany.

^2^The 1σ interval corresponds to the regional standard deviation of extinction in the respective zone, as can be obtained from the histogram of intensity values. This was also normalized to the reference time. Thus,

(9) $$\begin{array}{l} \sigma =\frac{{\langle \langle T^{-1}\left[o^{*},t_{i}\right]\rangle \rangle }^{\frac{1}{2}}}{\langle T^{-1}\left[o^{*},t_{0}\right]\rangle }. \end{array}$$

Since the experiment was performed once for each flow velocity, the error bars explicitly do not describe the standard deviation of the measurement, as can be obtained by repeating the same experiment several times.
