# Supplementary material for: Secretory Vesicle and Glucoamylase Distribution in Aspergillus niger and Macromorphology in Regions of Varying Shear Stress
Source: Front Microbiol. 2022 May 20;13:842249. doi: 10.3389/fmicb.2022.842249 (PMC9164161; doi:10.3389/fmicb.2022.842249)
Supplement: Supplementary file 1 [file Data_Sheet_1.PDF]

Supplementary Material

1 FIGURES

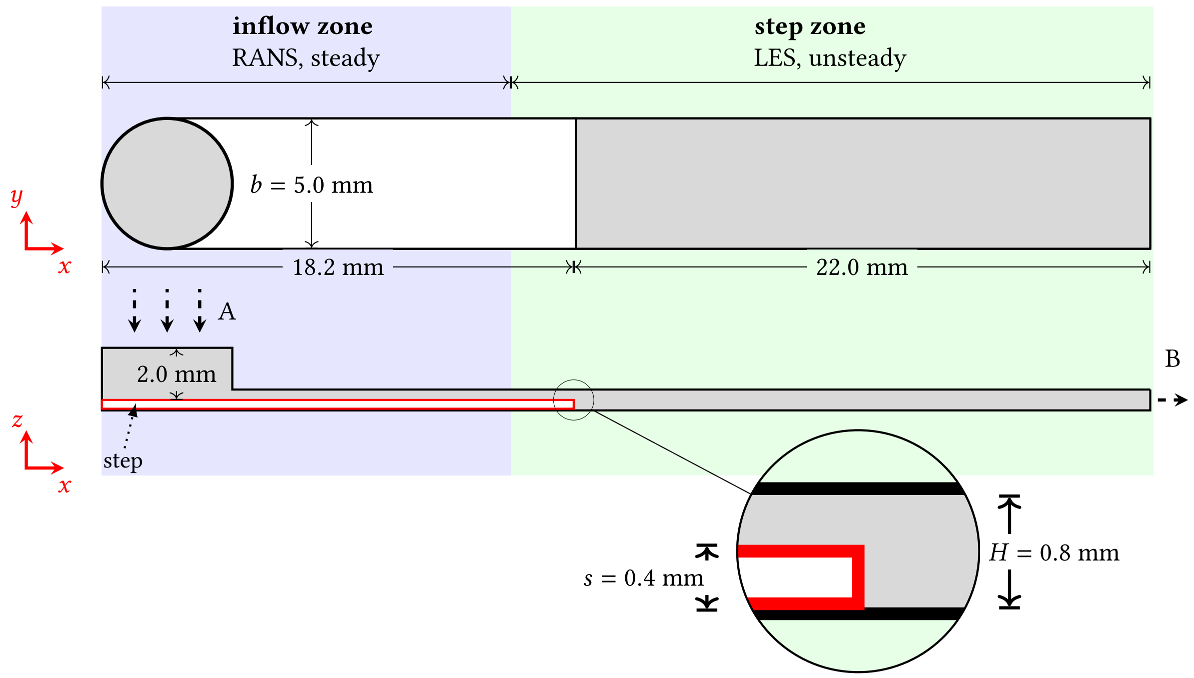

Figure S1. Interior of the step chamber, schematic diagram with dimensions. *Purple background*: inflow zone in front of the backward-facing step including Luer inlet port, plenum and settling zone in front of the step. *Green background*: step zone including the step and the wake zone. The velocity profile at the outlet of the inflow zone serves as a stationary inlet for the step zone. *Red*: taped-in step at the bottom of the step chamber, below the luer inlet port.

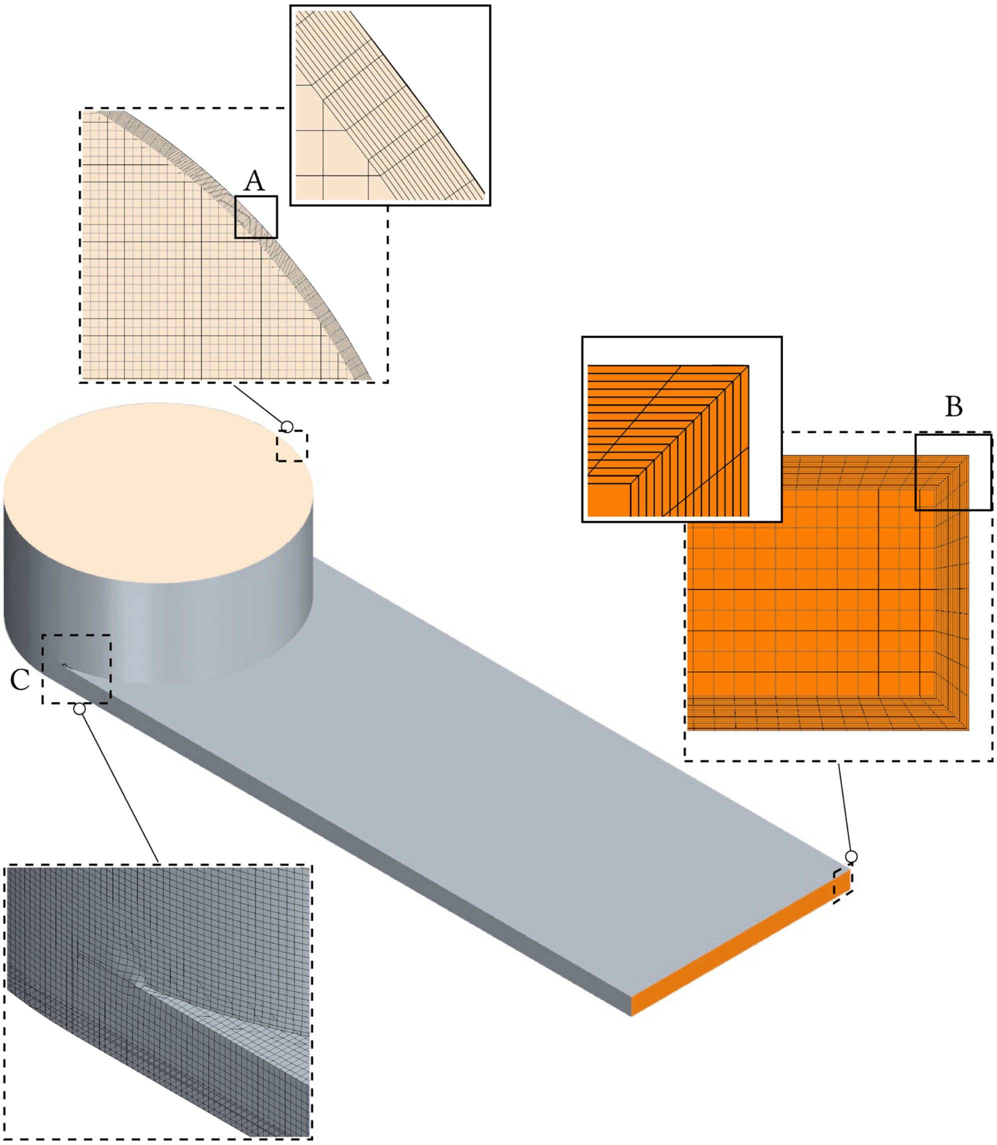

Figure S2. Three-dimensional representation of the computational grid of the inflow zone. Enlarged sections: A: prismatic layer at the edge of the inlet nozzle, B: prismatic layer at the edge of the main zone, C: external view of the tetrahedral grid at the transition of the inlet nozzle to the main zone.

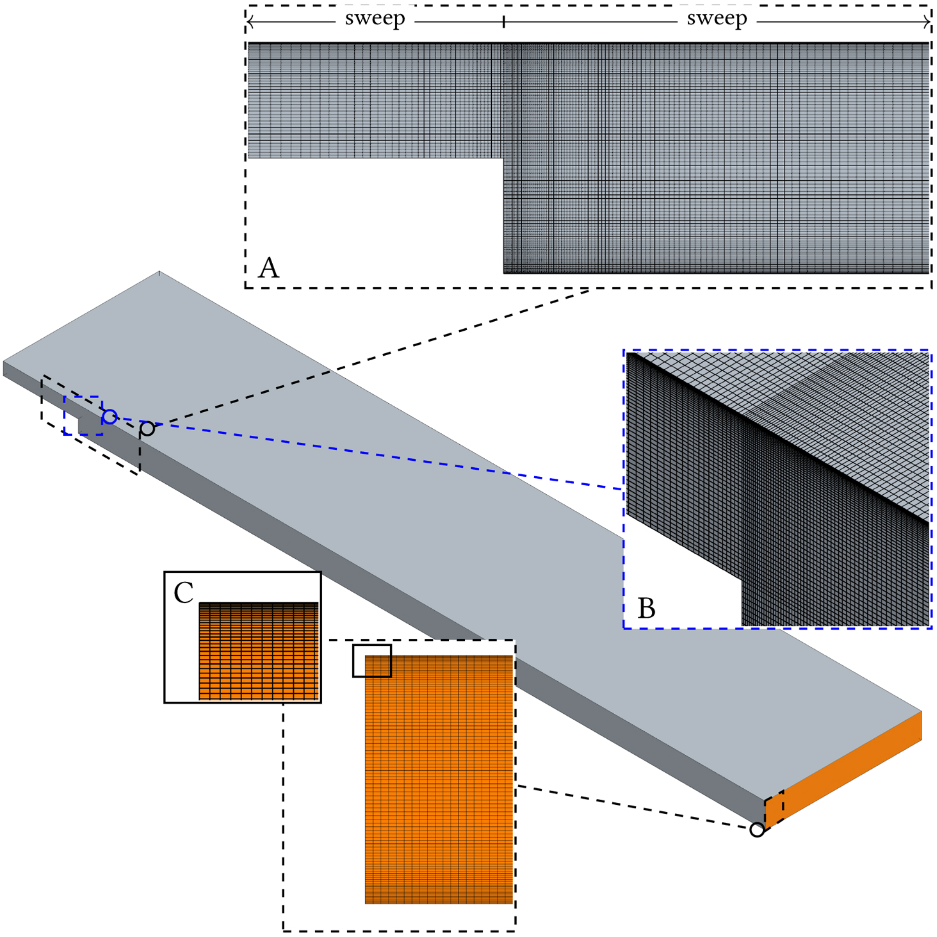

Figure S3. Three-dimensional representation of the computational grid of the step zone. Enlarged sections: A: side profile of the grid at the backward-facing step with `sweeping`, i.e. Spreading of the grid spacing, B: External view of the tetrahedral grid at the step, C: Profile view of the grid in the trailing zone with condensation of the grid to the horizontal surfaces.

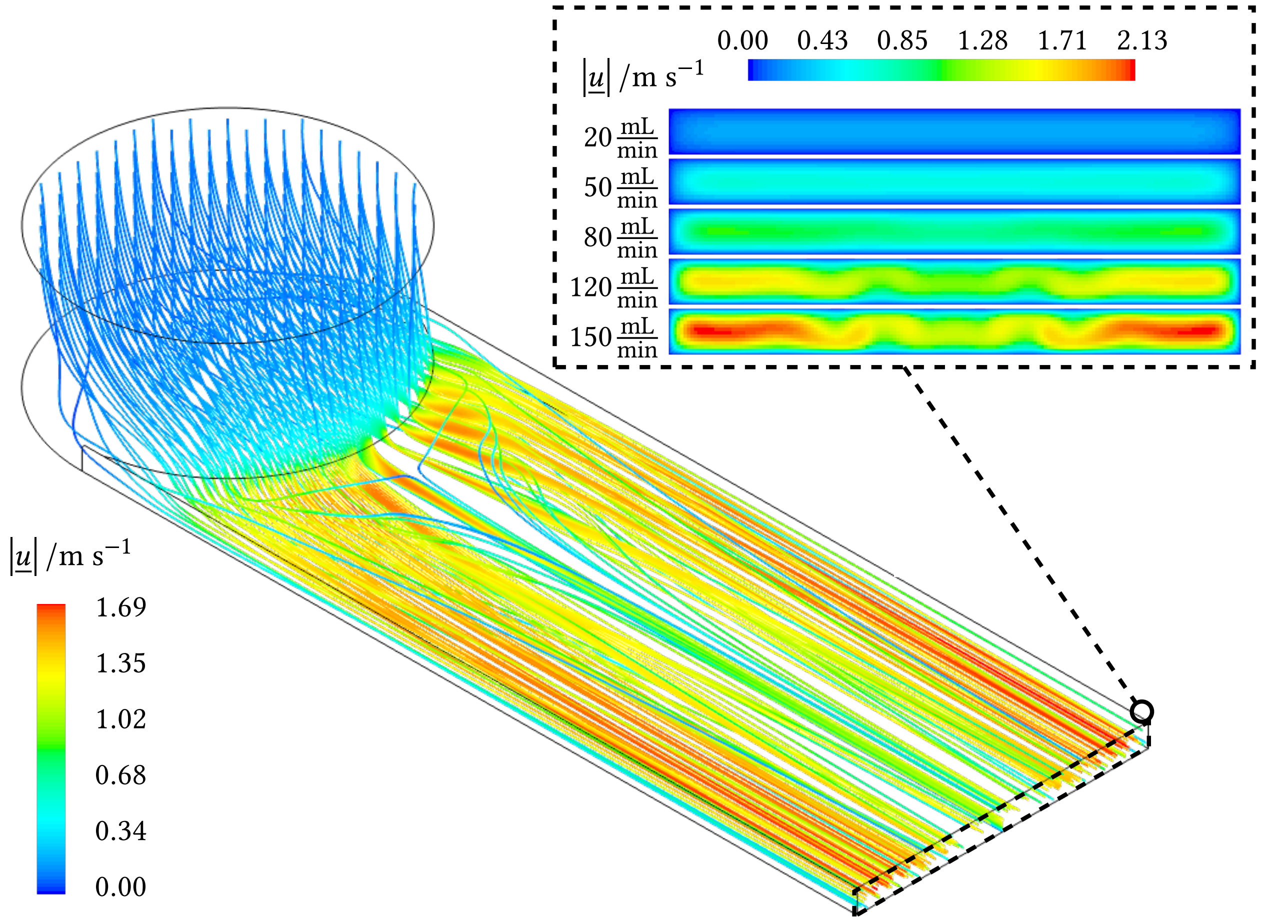

Figure S4. Result of the steady-state CFD simulation of the inflow zone (Figure S1, purple): streamlines starting from the Luer inlet for $\dot{V} = 120$ mL min^{-1}. *top right:* Velocity profiles ($|\underline{u}|$) at the outlet of the inflow zone for simulated volume flows $\dot{V} = \{20, \ldots, 150\}$ mL min^{-1}. The velocity is increasingly inhomogeneous with increasing volume flow. Only near the side walls, high flow velocities are formed. For the simulations, a simplified RANS-vortex model was used. The velocity profiles serve as an inlet boundary condition for the (main) step zone (Figure S1, green).

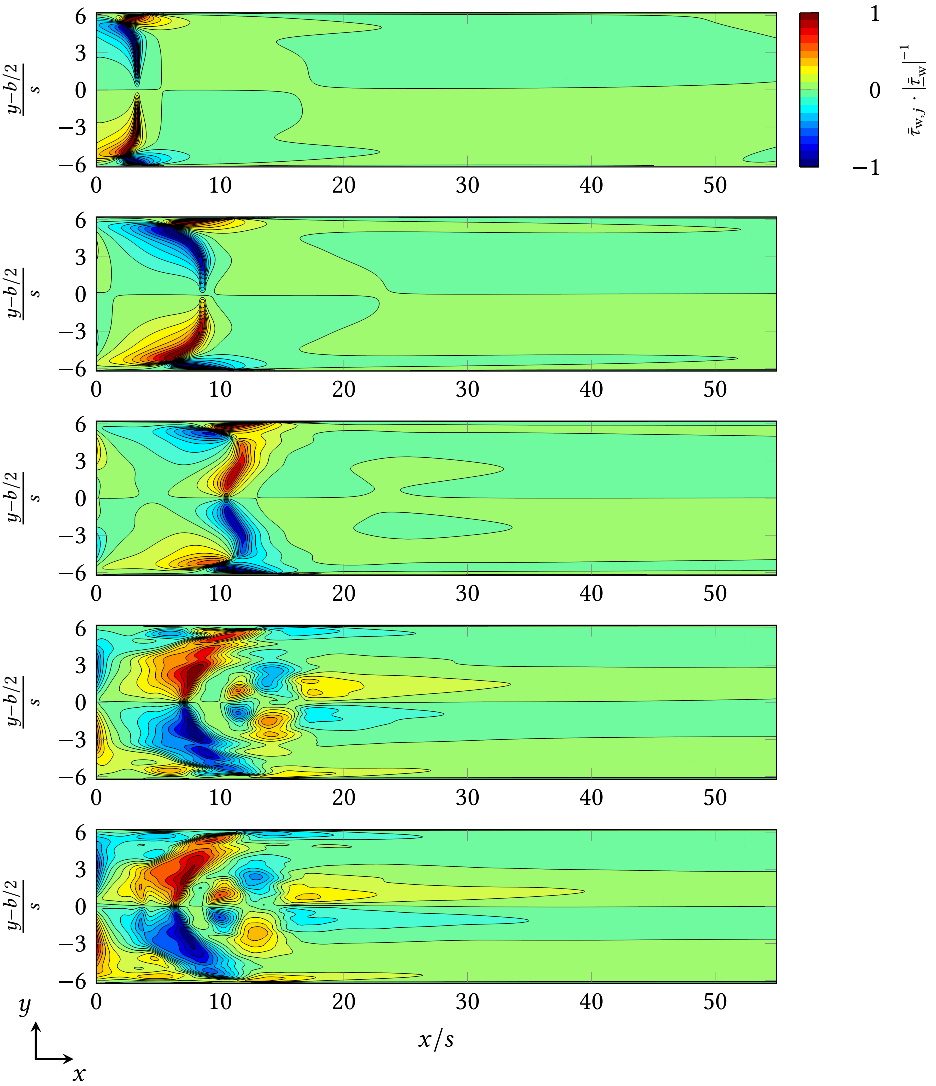

Figure S5. Relative proportion of y component of the wall shear stress $\bar{\tau}_{w,j}/|\underline{\bar{\tau}}_w|$ behind the backward-facing step for all $\dot{V} = \{20, 50, 80, 120, 150\}$ mL min^{-1} (top to bottom) in plan view. In the transient flow region above 80 mL min^{-1}, the fraction of sidestream flow – and hence its three-dimensionality – increases.

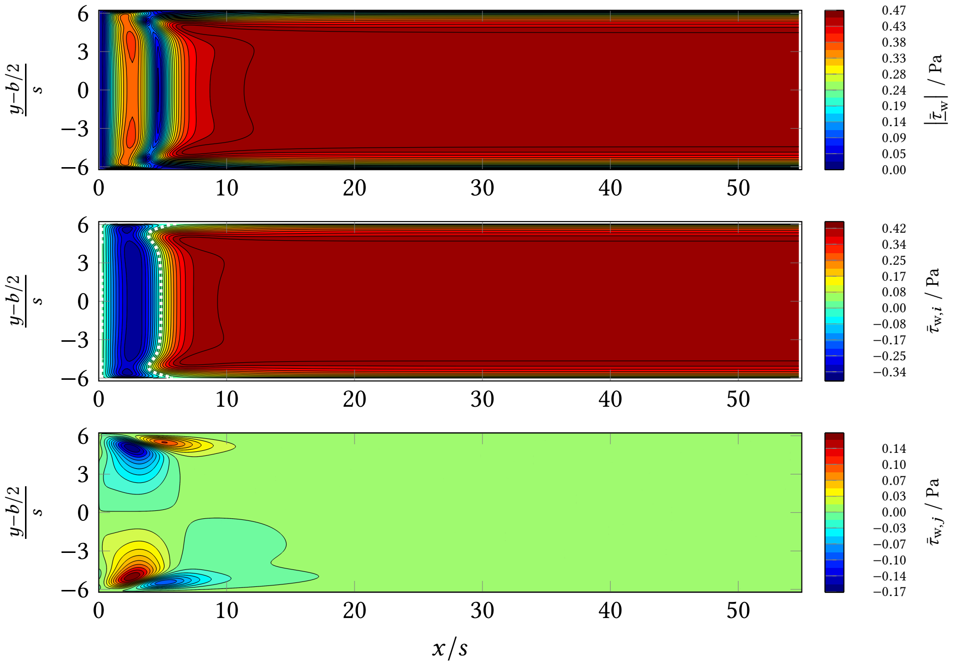

Figure S6. First statistical moment of the magnitude, x- and y-component of the wall shear stress ($\tau_{\mathrm{w},i}$, $\tau_{\mathrm{w},j}$, respectively) for $\dot{V} = 20$ mL min^{-1} .

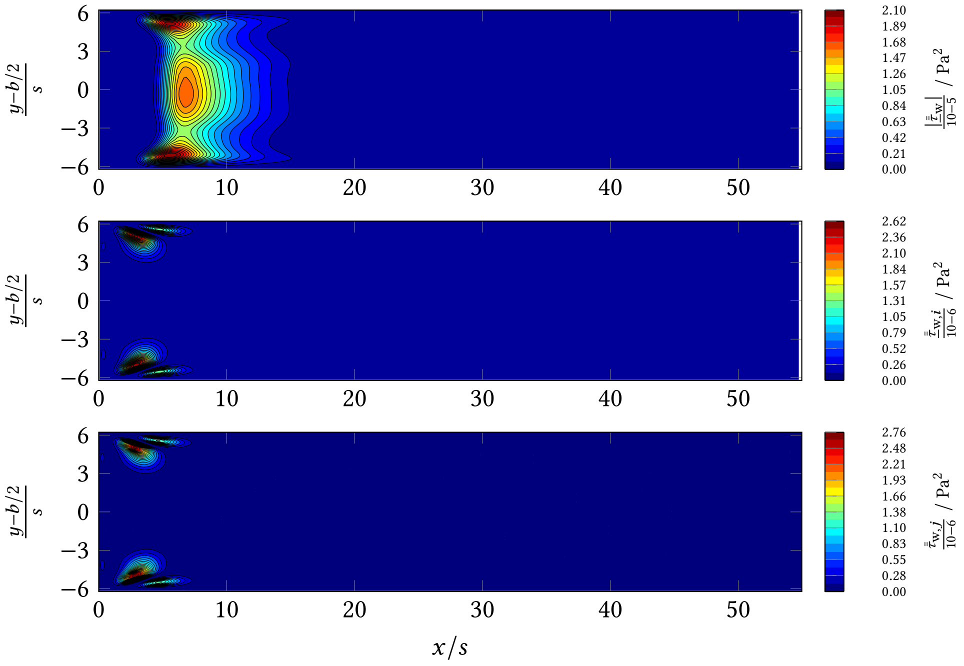

Figure S7. Second statistical moment of the magnitude, x- and y-component of the wall shear stress ($\bar{\bar{\tau}}_{\mathrm{w},i}$, $\bar{\bar{\tau}}_{\mathrm{w},j}$, respectively) for $\dot{V} = 20$ mL min^{-1} .

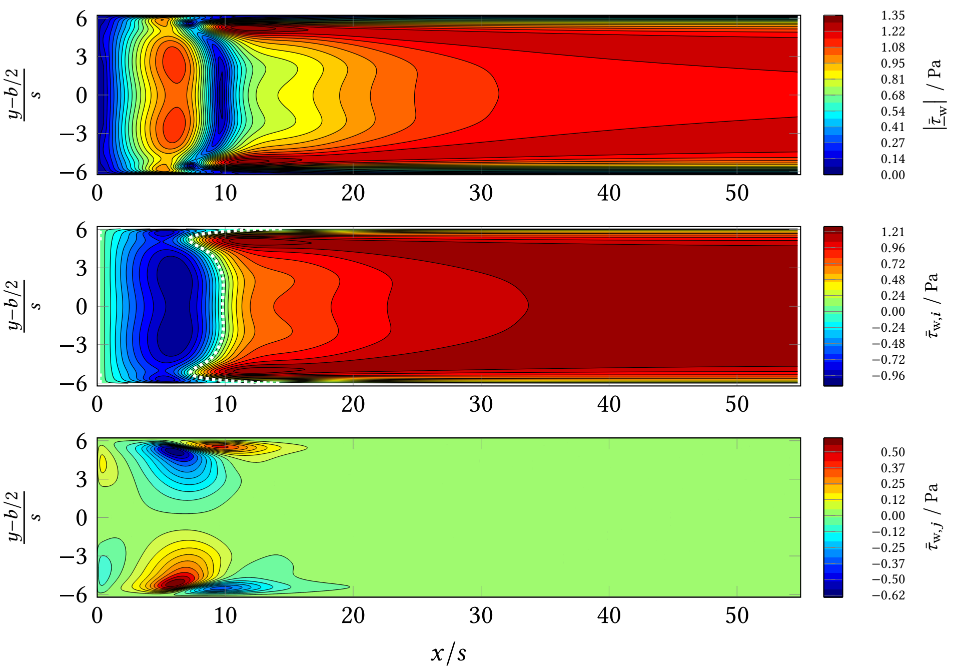

Figure S8. First statistical moment of the magnitude, x- and y-component of the wall shear stress ($\tau_{\mathrm{w},i}$, $\tau_{\mathrm{w},j}$, respectively) for $\dot{V} = 50$ mL min^{-1} .

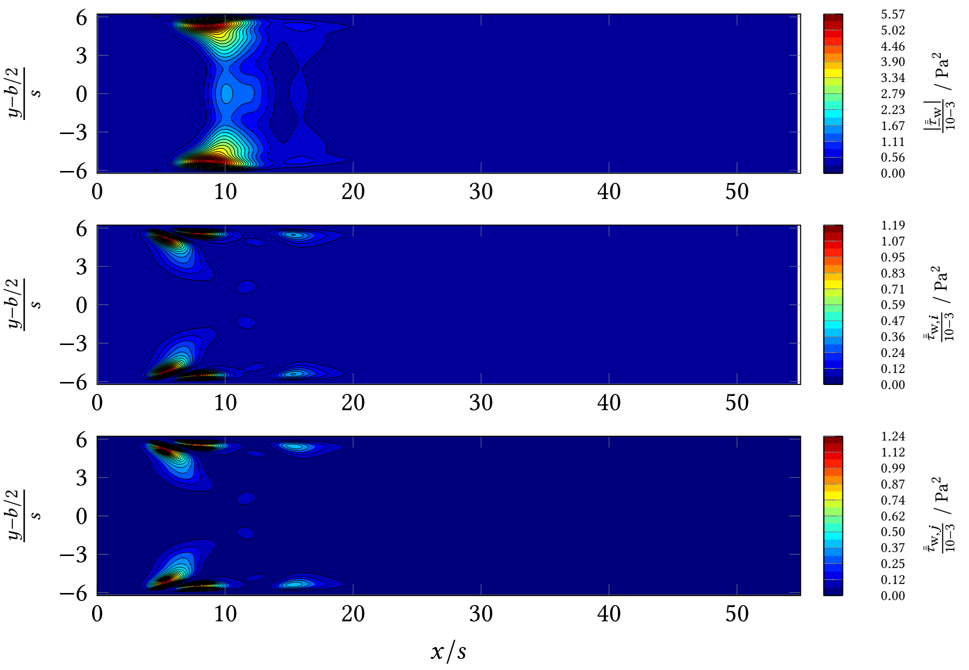

Figure S9. Second statistical moment of the magnitude, x- and y-component of the wall shear stress ($\overline{\overline{\tau}}_{\mathrm{w},i}$, $\overline{\overline{\tau}}_{\mathrm{w},j}$, respectively) for $\dot{V} = 50$ mL min^{-1} .

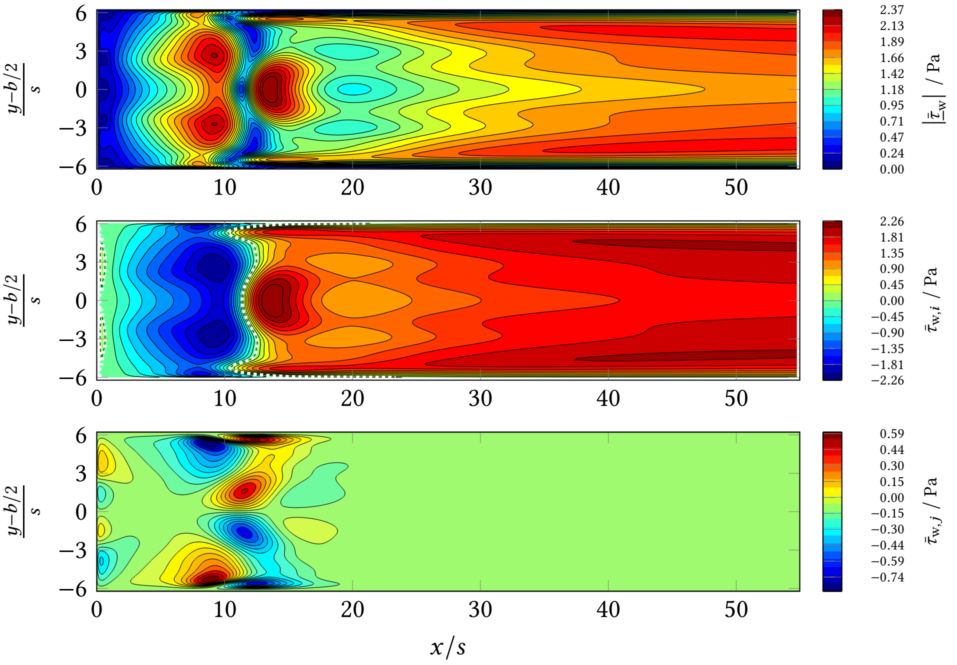

Figure S10. First statistical moment of the magnitude, x- and y-component of the wall shear stress ($\tau_{\mathrm{w},i}$, $\tau_{\mathrm{w},j}$, respectively) for $\dot{V} = 80$ mL min^{-1} .

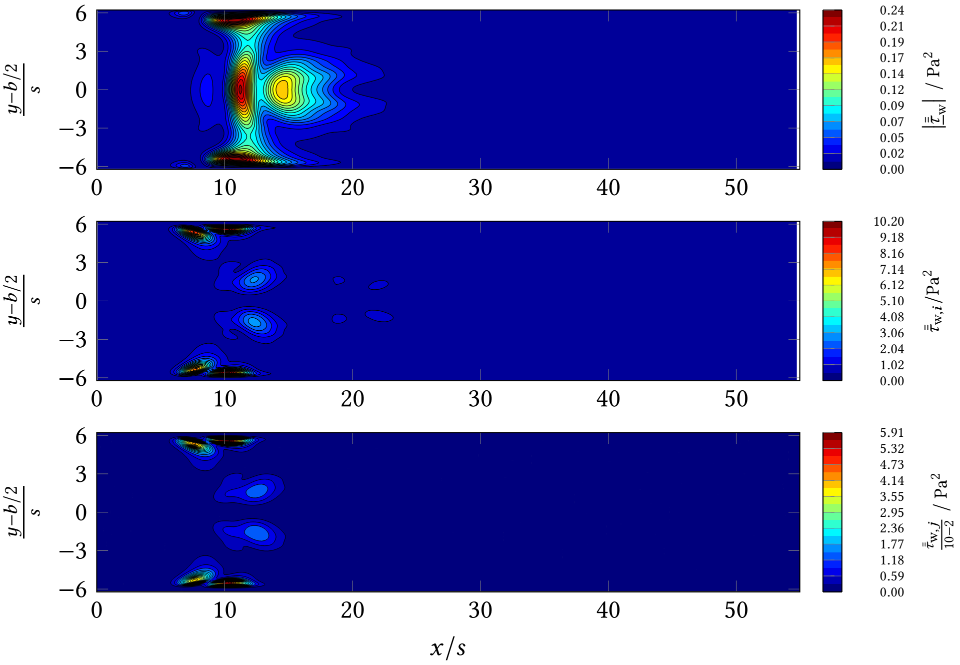

Figure S11. Second statistical moment of the magnitude, x- and y-component of the wall shear stress ($\bar{\bar{\underline{\tau}}}_{\mathrm{w},i}$, $\bar{\bar{\underline{\tau}}}_{\mathrm{w},j}$, respectively) for $\dot{V} = 80$ mL min^{-1} .

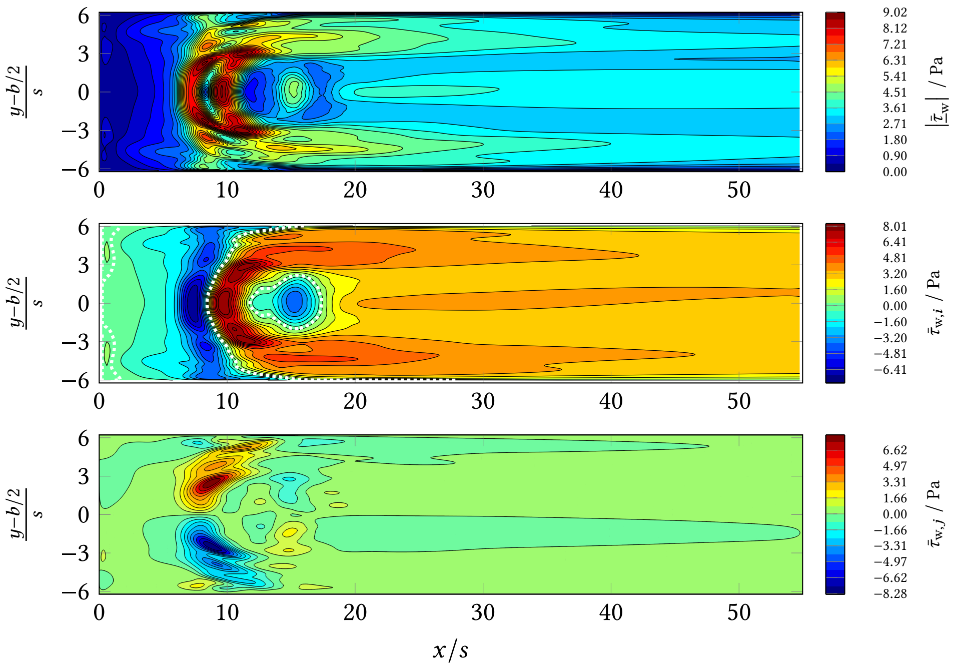

Figure S12. First statistical moment of the magnitude, x- and y-component of the wall shear stress ($\tau_{\mathrm{w},i}$, $\tau_{\mathrm{w},j}$, respectively) for $\dot{V} = 120$ mL min^{-1} .

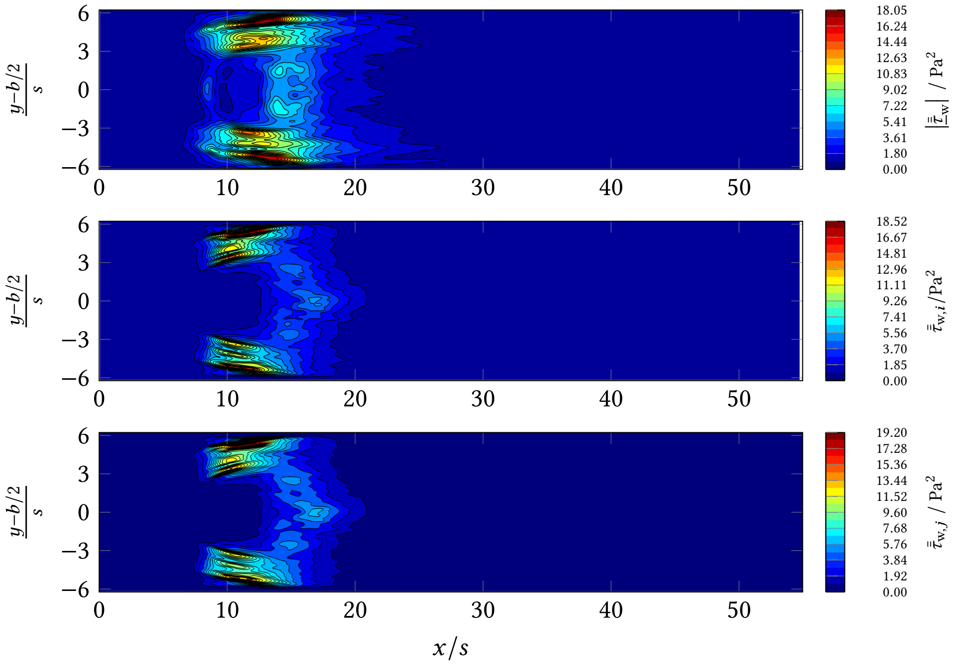

Figure S13. Second statistical moment of the magnitude, x- and y-component of the wall shear stress ($\bar{\bar{\tau}}_{\mathrm{w},i}$, $\bar{\bar{\tau}}_{\mathrm{w},j}$, respectively) for $\dot{V} = 120$ mL min^{-1} .

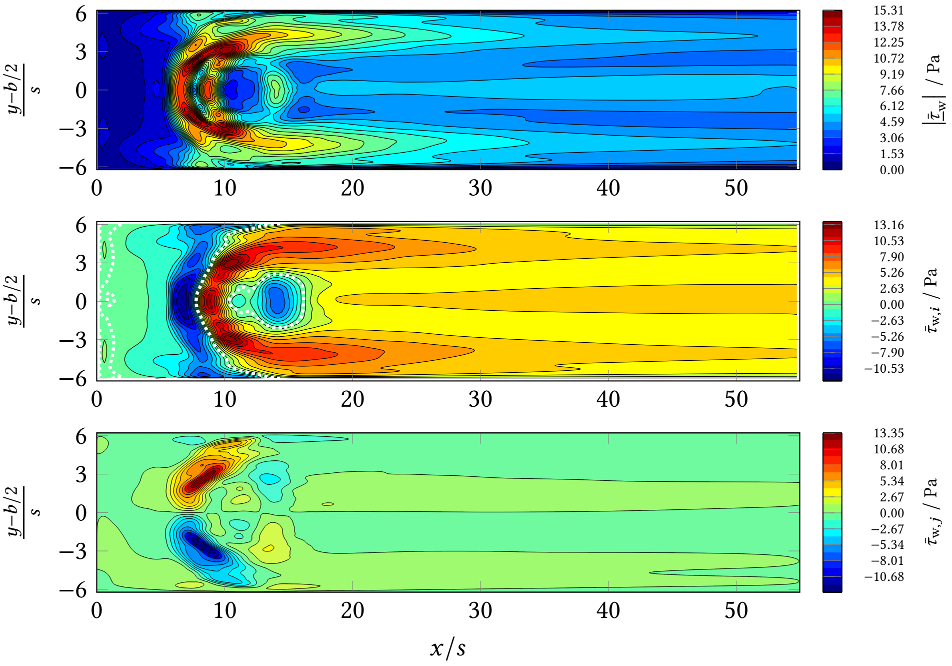

Figure S14. First statistical moment of the magnitude, x- and y-component of the wall shear stress ($\tau_{\mathrm{w},i}$, $\tau_{\mathrm{w},j}$, respectively) for $\dot{V} = 150$ mL min^{-1} .

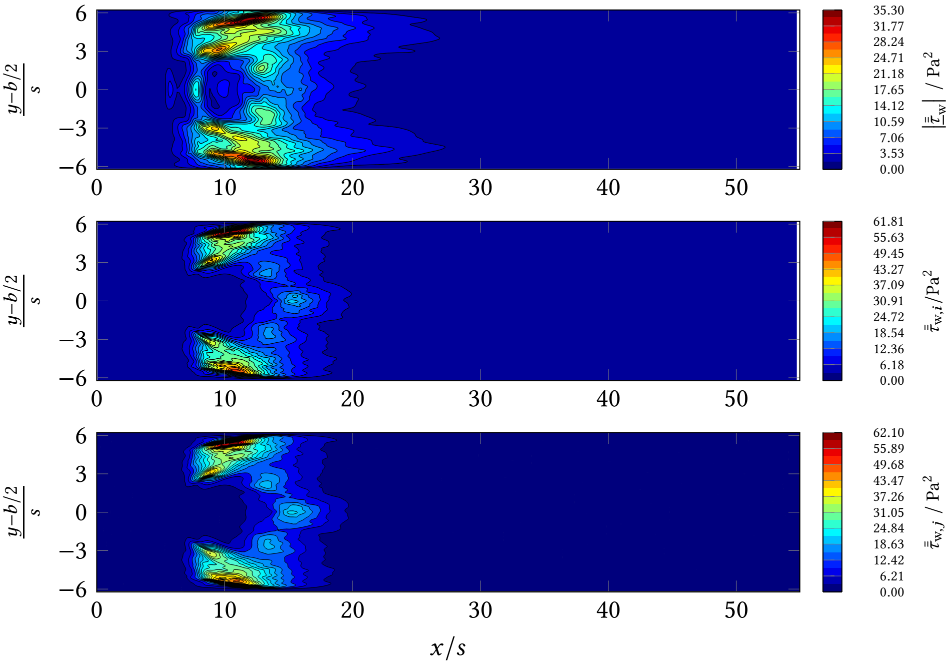

Figure S15. Second statistical moment of the magnitude, x- and y-component of the wall shear stress ($\bar{\bar{\tau}}_{\mathrm{w},i}$, $\bar{\bar{\tau}}_{\mathrm{w},j}$, respectively) for $\dot{V} = 150$ mL min^{-1} .

2 SETTINGS IMAGE ACQUISITION

Three-dimensional overview images were taken with the MF31.2 reporter strain in a total area of about 60 mm^2. The individual image data stacks were merged into an overall image in the LASX software (function: `tile merge`). An HL PL air lens with a lower magnification of 10×0.40 was used to acquire the individual images (1024×1024 Pxl). There were initially about $100 - 150$ spores in each area surveyed. Imaging settings on the CLSM are shown in Table S1 (*right column*).

Table S1. CLSM acquisition settings

spore density	100-150 spores / stack
no stacks	9×5 stacks form one image behind backward-facing step
image example	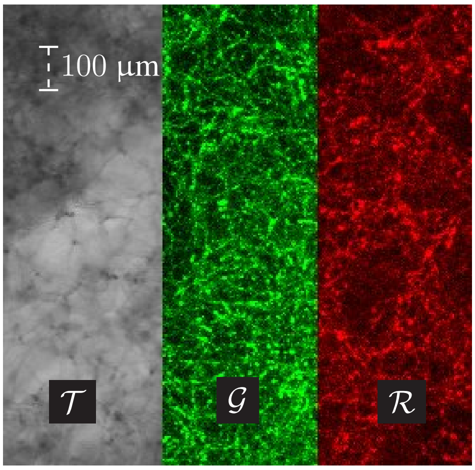
stack dimension	$\{\mathcal{T}, \mathcal{G}, \mathcal{R}\}\,[\mathtt{m}, \mathtt{n}, \mathtt{o}, \mathtt{t}_i]$
channels	transmission, green and red fluorescence
dimension of new image	$1550 \times 1500 \times 320$ µm^3
resolution	1024×1024 Pxl
$\Delta x/y; \Delta z$	1.5 µm Pxl^{-1}; 11.1 µm Vxl^{-1}
color space	$2^8 = 256$ gray values
objektiv	HC PL
magnification $\times$ num. aperture	10×0.40
immersion medium	air
laser	488 nm (blue)<br>552 nm (green)
detector range	490–547 nm (eGFP)<br>651–716 nm (dTomato)
